# Notes and News

**Published:** 1898-03-12

**Authors:** 


### Notes and News.

(Collected and Communicated.)

HOSPITAL MEETINGS.

North London Hospital for Consumption, Hampstead.—Annual Court of Governors, Wednesday, March 23rd, 1898, at 4 p.m., at 41, Fitzroy Square, W.

Mr. John Poland will lecture on "Deformities of the Bones after Injury," at the City Orthopædic Hospital, on Tuesday, March 15th, at 5.30 p.m.

Owing to the continuance of plague, steps are being taken by the Egyptian Government to prevent the usual pilgrimages to Mecca. In Bombay the plague continues to increase, and we regret to hear that, as last year, serious plague riots have occurred which have been attended by loss of life.

The citizens of Dundee commemorated the Diamond Jubilee by subscribing £44,000 towards a new Hospital for Incurables for their city. It is proposed to purchase Balgay House, a large mansion standing in five acres of land, and, by addition and rebuilding, to equip a hospital of 60 beds.

Mr. J. S. Risien Russell, M.D. (Edin), F.R.C.P., has been appointed assistant physician to University College Hospital. It is to be noted that in making this appointment the authorities have broken through the custom of restricting their choice to those educated at the Hospital, Dr. Charlton Bastian has been appointed consulting physician to the hospital, and the title of Emeritus Professor of Medicine and Clinical Medicine was conferred on him.

Dr. Archdall Reid advocates the "cage treatment" of surface wounds, *i.e.*, the wound is to be carefully cleansed twice a day or oftener, but beyond this nothing is to touch it but air. Under this treatment it is claimed that wounds heal "with a quickness as delightful to the surgeon as to the patient," and that the cicatrices which are produced are of a better character than those which follow the use of "dressings." This sounds like the "oxygen treatment" without the oxygen. Can it be, after all, that it is not the oxygen, but the absence of the irritation of dressings that enabled Mr. Stoker to obtain his success? There is a little tale about the making of stone broth. The receipt ran thus: "Take a pebble from the brook, boil it in spring water, adding from time to time pepper and salt and herbs to flavour and a little meat." We believe the stone broth was very good, and we have already given our testimony to the success of the oxygen treatment.

We feel rather sorry than otherwise to hear that a Mr. Wadlow, an unqualified assistant to a medical man, has been fined, along with his master, for non-notification of a case of scarlet fever. The imposition of a fine for the non-performance of a duty which can only be properly performed by a medical man seems to us a semi-recognition of the power of an unqualified assistant to sign certificates, which is much to be regretted.

Dr. Schenk, of Vienna, who some time ago put forward a theory as to the causes determining the sex of children, seems from all accounts to be having an unhappy time of it. It is said that every mail brings him multitudes of letters from anxious women all over the globe asking how they can bring about the conception of a male child. The gossips even say that certain royal houses which are "bothered with girls" have consulted him as to the diet which will produce a boy. Meanwhile, Dr. Schenk declares that his interest in the matter is purely scientific, and that he has never undertaken the professional treatment of such aspirants for male descendants. The public, however, does not care a fig for either science or scientists unless some practical result can be shown—in this case, a boy!

The Royal College of Physicians of London is an institution which consists of the Fellows thereof, and the sooner the dissatisfied members who imagine that they have any rights beyond those of having "the use of the library and museum, subject to the bye-laws and regulations relating thereto," and being "admitted to all lectures," the better for their peace of mind. It is a ridiculous position, but there it is. The membership is merely a permission for a man to stand upon the steps until the Fellows choose to let him in, while the Licentiates are, of course, quite outside the outer gates. Wherein lies the joy of being a Fellow is perhaps a mystery; perhaps, indeed, beyond the pleasure of possessing what others want, but have not got, there is not much in it. But so long as the Fellows hold the key we would mildly suggest to those members who are so anxious for admission that all their "dissatisfaction" with respect to the elections to the Fellowship is but sweet incense offered to the gods.

A question of great interest arises at the present day in regard to the isolation hospitals which are springing up all over the country, viz., Are they being put up under a false pretence? If these hospitals, in which people are being treated free, gratis, and for nothing, are being put up openly and honestly because, as is insisted on by Dr. Meredith Young in a letter to the *Lancet*, it pays the community better to have people well treated in hospital than to leave them in their homes to be added to the chronic invalids of the population and drift gradually into pauperism, all well and good. But there can be no doubt that in most cases they are being erected under the impression that their object is to limit the spread of epidemics. These are two quite different things. We think that those who advocate the free admission of all without fee to such hospitals should make up their minds as to the grounds on which they base their recommendations. If they regard hospitals as a means of preventing epidemics, they will probably receive general support; but to use them for the prevention of chronic illness is another affair altogether, and any argument based upon the utility of isolation hospitals for such a purpose would equally apply to hospitals of all kinds. We hardly think that the sanitary authorities, even though incited thereto by the most plausible of medical officers of health, would be ready to take up the gratuitous treatment of all kinds of illness in all conditions of men.

